# Transcriptome analysis provides genome annotation and expression profiles in the central nervous system of *Lymnaea stagnalis* at different ages

**DOI:** 10.1186/s12864-021-07946-y

**Published:** 2021-09-03

**Authors:** Martina Rosato, Brittany Hoelscher, Zhenguo Lin, Chidera Agwu, Fenglian Xu

**Affiliations:** 1 Department of Biology, College of Arts and Sciences, Saint Louis University, St. Louis, MO USA; 2 Henry and Amelia Nasrallah Center for Neuroscience, Saint Louis University, St. Louis, MO USA; 3 Department of Pharmacology and Physiology, Saint Louis University, School of Medicine, St. Louis, MO USA

**Keywords:** *Lymnaea stagnalis*, CNS, Transcriptome, Gene annotation, Different ages, Neurodevelopment, Synaptic genes, Disease-related genes

## Abstract

**Background:**

The pond snail, *Lymnaea stagnalis* (*L. stagnalis*), has served as a valuable model organism for neurobiology studies due to its simple and easily accessible central nervous system (CNS). *L. stagnalis* has been widely used to study neuronal networks and recently gained popularity for study of aging and neurodegenerative diseases. However, previous transcriptome studies of *L. stagnalis* CNS have been exclusively carried out on adult *L. stagnalis* only. As part of our ongoing effort studying *L. stagnalis* neuronal growth and connectivity at various developmental stages, we provide the first age-specific transcriptome analysis and gene annotation of young (3 months), adult (6 months), and old (18 months) *L. stagnalis* CNS.

**Results:**

Using the above three age cohorts, our study generated 55–69 millions of 150 bp paired-end RNA sequencing reads using the Illumina NovaSeq 6000 platform. Of these reads, ~ 74% were successfully mapped to the reference genome of *L. stagnalis*. Our reference-based transcriptome assembly predicted 42,478 gene loci, of which 37,661 genes encode coding sequences (CDS) of at least 100 codons. In addition, we provide gene annotations using Blast2GO and functional annotations using Pfam for ~ 95% of these sequences, contributing to the largest number of annotated genes in *L. stagnalis* CNS so far. Moreover, among 242 previously cloned *L. stagnalis* genes, we were able to match ~ 87% of them in our transcriptome assembly, indicating a high percentage of gene coverage. The expressional differences for innexins, FMRFamide, and molluscan insulin peptide genes were validated by real-time qPCR. Lastly, our transcriptomic analyses revealed distinct, age-specific gene clusters, differentially expressed genes, and enriched pathways in young, adult, and old CNS. More specifically, our data show significant changes in expression of critical genes involved in transcription factors, metabolisms (e.g. cytochrome P450), extracellular matrix constituent, and signaling receptor and transduction (e.g. receptors for acetylcholine, N-Methyl-D-aspartic acid, and serotonin), as well as stress- and disease-related genes in young compared to either adult or old snails.

**Conclusions:**

Together, these datasets are the largest and most updated *L. stagnalis* CNS transcriptomes, which will serve as a resource for future molecular studies and functional annotation of transcripts and genes in *L. stagnalis*.

**Supplementary Information:**

The online version contains supplementary material available at 10.1186/s12864-021-07946-y.

## Background

The pond snail *Lymnaea stagnalis (L. stagnalis)* belongs to the phylum Mollusca, class Gastropoda [1]. Like its counterpart, the sea slug *Aplysia californica (A. californica)*, *L. stagnalis* has served as an important mollusc model organism for the neurobiology field since the 1970s due to its simple central nervous system (CNS), well characterized network, large and easily accessible neurons, and a relatively short life cycle [2]. *L. stagnalis* CNS contains a total of 20,000–25,000 neurons organized in a ring of 11 connected ganglia. The neurons are large in size (up to ~ 100 μm in diameter versus ~ 15–25 μm in diameter of vertebrate central neurons) [2, 3] and easily recognizable, making them a perfect target for in vitro and in vivo studies. Many studies have used this model to investigate the fundamental mechanisms of neuronal networks involved in various behaviours including feeding [4, 5], respiration [6, 7], locomotion [8, 9], and reproduction [10, 11]. Studies have also focused on high cognitive behaviours, including learning and memory [12–15], as well as deciphering cellular mechanisms of synapse formation and synaptic plasticity during development [16–18]. *L. stagnalis* has also recently gained increasing popularity for the investigation of brain aging and neurodegenerative diseases such as Parkinson’s disease and Alzheimer’s disease [19–23]. It is important to note that comparative studies have highlighted several human homologs involved in aging and neurodegenerative disease in both *A. californica* and *L. stagnalis* [24–26], showing the great potential for future molecular insights into brain aging and pathology using these unique mollusc models. More importantly, a recent study has successfully established the use of CRISPR/Cas9 in *L. stagnalis* embryos [27], further underscoring the high feasibility of *L. stagnalis* for genetic studies.

Despite the importance of *L. stagnalis* to brain network, behaviour, and development studies, genetic information is mostly limited to the identification and cloning of individual genes. Only in the past decade several groups have tried to study the *L. stagnalis* transcriptome using expressed sequence tags (EST) [28–30] and RNA sequencing (RNA-Seq) [31, 32]*.* However, all these studies have focused on only one developmental time point, predominantly in adults. Considering the increasing use of *L. stagnalis* for brain aging and pathology research [24], updated transcriptome datasets and gene annotations including old or aging snail CNS are critically needed. Studies of transcriptional changes in brains of animals at various ages will provide important molecular insights into brain development, aging, pathology, and evolution. Spatial and/or temporal transcriptome analyses of brains and other tissues have been carried out in human [33, 34], rats [35], mice [36], chicken [37], zebrafish [38], and birds [39] among others. All these studies have contributed to our understanding of the molecular basis of brain development. Invertebrates have also been utilized for study of development and aging. Developmental transcriptomes of well-established invertebrate models such as *Caenorhabditis elegans* (*C. elegans*) [40, 41] and *Drosophila melanogaster* (*D. melanogaster*) [42] have been reported. Recent efforts have also focused on the transcriptome of aging *D. melanogaster* [43, 44] and *C. elegans* [45], aiming to reveal molecular mechanisms of longevity or aging trajectories.

In mollusca, the developmental (embryonic, larval, and metamorphic) transcriptome of *A. californica* [46] and maternal (1 to 2 cell and ~ 32 cell) transcriptome of *L. stagnalis* have been conducted [47]. These studies shed novel insights into conserved sets of genes and pathways in early development. However, these studies failed to inform how these genes or other sets of genes are regulated in later stages of life, such as after animals are fully matured and aged. Although transcriptome changes in a subset of sensory neurons involved in tail-withdrawal reflex in matured and aged *A. californica* have been reported [48], transcriptome changes of entire CNS in young, mature, and aged *A. californica* and *L. stagnalis* have not been carried out. Understanding transcriptome changes in the entire CNS, but not a confined set of neurons, will shed light in the overall genetic profiles for nervous system development and aging, as well as for regulation of all ranges of animal behaviours. Such studies are critical for our complete understanding and comparative studies of age- or species- specific molecular strategies that are key to the evolution, survival, and function of both invertebrate and vertebrate.

To this end, in the present study, we provide whole transcriptome analysis in *L. stagnalis* CNS from three different ages: 3 months (young), 6 months (adult), and 18 months (old). This is the first time that changes in CNS transcriptome profiling during brain development, maturation, and aging in *L. stagnalis* are analyzed. Our RNA-Seq, using Illumina NovaSeq 6000 platform, produced 56–69 millions of 150 bp paired-end reads, and 74% of these reads were mapped to the draft genome of *L. stagnalis*. We provide gene annotations for 32,288 coding sequences with a minimum of 100 codons, contributing to the largest number of annotated genes for the *L. stagnalis* genome to date. Furthermore, our data reveal age-specific, differentially expressed genes and enriched pathways in young, adult, and old CNS. Among those, we have highlighted genes implicated in neural development and synaptic function, as well as stress and disease conditions.

## Results

### *L. stagnalis* CNS transcriptome sequencing, assembly, and gene annotation

**Fig. 1:**
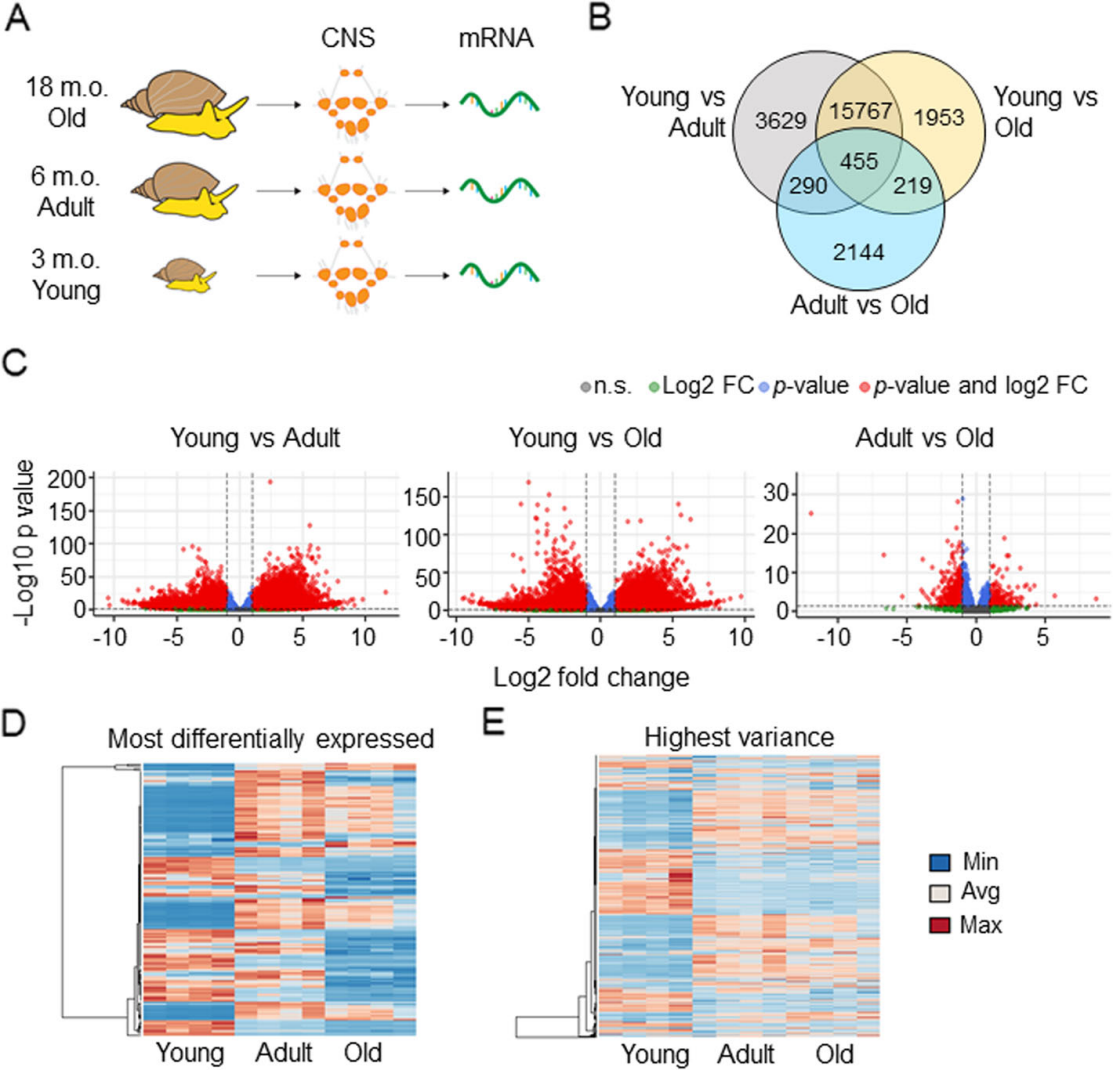
Transcriptomes pairwise comparisons reveal a specific pattern of gene expression in *L. stagnalis* CNS. **A** For transcriptome analysis, mRNA was extracted from the CNS of young (3 months old), adult (6 months old), and old (18 months old) snails. For each age group, four different biological replicates (*n* = 4), each with mRNA samples from the CNS of 10 snails, were used. **B** Venn diagram showing the significantly differentially expressed genes in each pairwise comparison and the overlap among them. The diagram clearly shows that young CNS transcriptome has more significantly differentially expressed genes compared to adult and old. (FDR adjusted *p*-value *p* < 0.05; log2 fold change > |1|). **C** Volcano plot of each pairwise comparison. The plots are color-coded based on the log2 fold change (green dots), and corrected *p*-value (blue dots). Red dots highlight genes that are significant and whose expression is highly changed (FDR adjusted *p*-value *p* < 0.05; log2 fold change > |1|). Volcano plots of the comparison between adult and old CNS transcriptome shows less differentially expressed genes, by either *p*-value or log2 fold-change, compared to the pairwise comparison of young versus adult or old transcriptome. **D**, **E** Heatmap of the most differentially expressed genes in all pairwise comparisons (FDR adjusted *p*-value *p* < 0.05) and the genes with the highest variance (top 2000 genes), respectively. Both heatmaps show a distinct pattern of gene expression in the CNS transcriptome of young snails compared to adult and old

RNA-Seq was performed using CNS samples from young (3 months old), adult (6 months old), and old (18 months old) snails (Fig. 1A), with four biological replicates in each group and ten snails in each replicate. *L. stagnalis* has a relatively short life cycle, with a life expectancy of about 1.5 to 3 years [49]. The embryonic stage of the snail lasts for around 2 weeks, and eggs are contained in gelatinous masses that are accessible for genetic manipulation. After hatching, young snails reach sexual maturity at around 4 to 6 months of age, and senescence starts after 7–8 months [49, 50]. Therefore, the 3-month-old age in our study represents a rapid developing and sexual immature stage, the 6-month-old age represents a fully, sexually mature stage, and 18-month-old represents an aging stage [49, 50].

Our RNA-Seq data provides a good sequencing depth, with a total number of reads ranging from 55,601,129 (56 M) to 69,121,300 (69 M) and an average overall alignment rate is ~ 74% (Table 1). A total of 61,994 transcripts from 42,478 genes are identified. Of those genes, 37661 encode for proteins of at least 100 amino acids. To provide functional annotations for inferred *L. stagnalis* genes, we retrieved proteomic sequences of nine molluscan species from the NCBI RefSeq database (See Methods). Our transcript assembly and gene function annotation provide the first genome annotation for *L. stagnalis* with 32,182 out of 33,786 unique transcripts (95.25%) that have matched Pfam domain and/or homologs (provided as Additional file 2 in gff3 format).

**Table 1:** Mapping summary of RNA-Seq data

Sample ID	Raw reads	Pairs	overall alignment rate	aligned concordantly exactly 1 time
A1	111,202,258	55,601,129	73.64%	32,393,971 (58.26%)
A2	117,009,046	58,504,523	70.58%	32,531,948 (55.61%)
A3	126,008,938	63,004,469	68.96%	33,453,481 (53.10%)
A4	118,371,182	59,185,591	66.10%	29,961,867 (50.62%)
A5	120,837,330	60,418,665	77.56%	37,389,888 (61.88%)
A6	130,596,268	65,298,134	78.03%	40,020,465 (61.29%)
A7	138,242,600	69,121,300	76.80%	41,408,979 (59.91%)
A8	120,985,900	60,492,950	77.88%	36,964,875 (61.11%)
A9	114,785,564	57,392,782	76.97%	34,327,672 (59.81%)
A10	111,292,186	55,646,093	76.08%	33,245,908 (59.75%)
A11	125,842,254	62,921,127	77.27%	38,379,882 (61.00%)
A12	120,566,284	60,283,142	75.40%	35,319,113 (58.59%)

### Transcriptional clustering pattern in the CNS of young, adult, and old *L. stagnalis*

Our principal component analysis (PCA) of log2 of the raw counts for the 12 transcriptomes (three age groups, four replication samples per age group) form three major clusters (Additional Figure 1A), corresponding to the three age groups of samples. The majority of biological replicates cluster together, suggesting that expression profiles are more similar in animals belonging to the same-age cohort. The first principal component (PC1), which accounts for 68.12% of the variance in the data, provides separation between young and the other two groups (adult and old). The second principal component (PC2) only accounts for 7% of the variance, serving as a discriminator between adult and old transcriptomes. These patterns suggest that there are constitutive differences in transcriptomes between young and adult/old CNS, while adult and old CNS transcriptomes are more similar to each other. These results are consistent with pairwise Pearson correlations between these samples (Additional Fig. 1B**)**.

To identify genes that are differentially expressed (DE) during the development of the CNS, we conducted pairwise comparisons of transcriptomes for the three groups of samples: young vs. adult, young vs. old, and adult vs. old. We identify 20,141 significant DE genes between young and adult groups; 18,394 DE genes between young and old groups; and only 3108 significant DE genes between adult and old groups (FDR adjusted *p*-value *p* < 0.05; log2 fold change > |1|) (Fig. 1B,C). Interestingly, only 455 DE genes are present in all comparisons. Together, these analyses suggest that most changes in CNS development occur pronouncedly during transitions from young to adult, and less changes occur during transitions from adult to old.

### Analysis of the DE genes confirms distinct gene expression patterns in young, adult, and old *L. stagnalis*

Next, we selected the top 100 genes that, among those sequences with at least 100 codons, are the most DE based on their adjusted *p*-values in each pairwise comparison. After assessing a partial overlap between each pairwise comparison, a total of 143 unique most DE genes are used. Heatmap analysis of FPKM expression shows a different expression pattern among age groups (Figs. 1D,E). Specifically, consistent with the above PCA, individual replicates exhibit very similar regulation patterns within the same age cohort (Fig. 1D). Overall, the adult animal transcriptome shows more highly expressed genes, while around half of genes in young and the majority of genes in old exhibit low expression. The heatmap pattern also shows that 1) most genes with low expression in young animals are highly expressed in adult and remain high in old; 2) most highly expressed genes in young animals have low expression in adult and become further down-regulated in old animals; 3) only a few clusters contain genes whose expression increases from young to adult and then decreases from adult to old (Fig. 1D). We also looked at the top 2000 genes with the highest variance across all samples. The heatmap in Fig. 1E shows 1) variance in DE gene expression, again, separates young transcriptome from transcriptomes of adult and old, and the highest variance occurs in the young animal transcriptome; 2) two big clusters of genes increase in expression from young to adult CNS transcriptome and retain relatively high expression in old; 3) two big clusters of genes decrease in expression from young to adult CNS transcriptome and further lower their expression in old; 3) a few small clusters of genes increase expression from young to adult and then decrease from adult to old. Together, these data indicate that there are distinct changes of transcript profiling across life stages of animals. Next, we sought to study what sets of DE genes and related pathways are involved in the expression patterns across different life stages.

### Gene ontology analysis

**Fig. 2:**
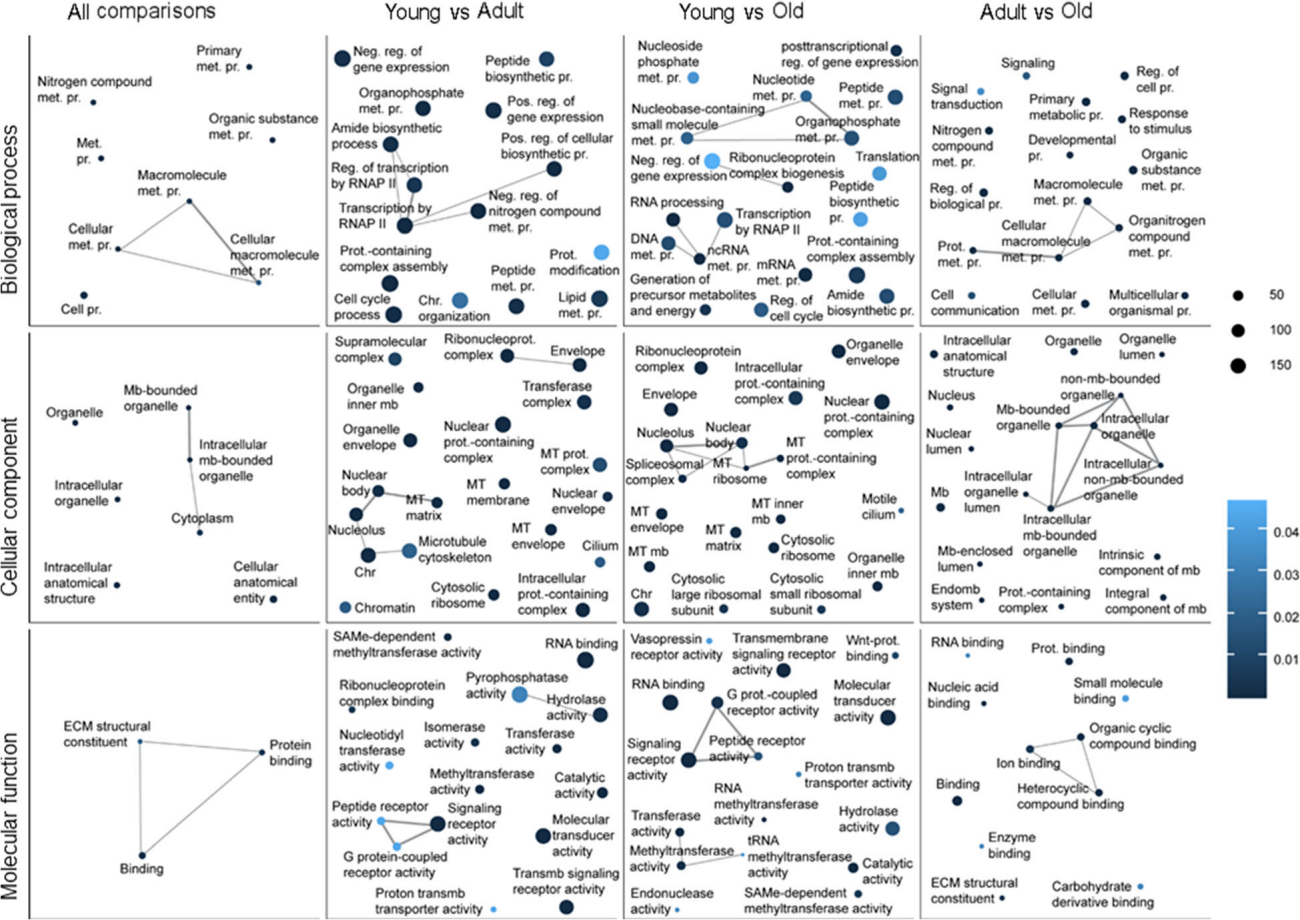
Gene Ontology (GO) enrichment analysis of differentially expressed genes. Representative enriched GO terms are shown as dots for each GO category (Biological process, Cellular component, or Molecular function). The significantly differentially expressed genes in all pairwise comparisons (All comparisons, 455 genes), in the comparison between young and adult CNS transcriptomes (Young vs. Adult, 20,141 genes), young and old CNS transcriptomes (Young vs. Old, 18,394 genes), or adult and old CNS transcriptomes (Adult vs Old, 3108 genes) were used for the analysis. The dot size reflects the number of genes in the GO term for each significantly differentially expressed gene set; the dot colour represents the FDR-corrected *p*-value, with darker colours indicating lower *p*-values; the line size represents the degree of similarity. Abbreviations: met. = metabolic, pr. = process, neg. = negative, pos. = positive, reg. = regulation, mb = membrane, prot. = protein, chr = chromosome, RNAP II = RNA polymerase II, MT = mitochondrial, ECM = extracellular matrix

We performed GO annotation with Blast2GO [51] for the 37,661 transcripts that encode proteins with at least 100 amino acids (see Methods). A total of 32,288 transcripts (or genes) were successfully annotated with GO terms. GO enrichment analysis was performed for DE genes in: all pairwise comparisons (455 genes), young compared to adult CNS transcriptome (20,141), young compared to old CNS transcriptome (18,394), and adult compared to old CNS transcriptome (3108) (FDR adjusted *p*-value *p* < 0.05; log2 fold change > |1|) (Fig. 2). As expected from the huge overlap among significant DE genes, GO enrichment from young compared to adult and young compared to old groups partially overlap (Fig. 2). In these two comparisons, the common GO terms in biological process are related to gene expression (transcription by RNA polymerase II and positive or negative regulation of gene expression). Genes in this pathway include the Rho GTPases cdc42 and tyrosine-protein kinase SRK2-like and several transcription factors, such as Sox14, Dp1, Sp4, and NF-κB. GO terms in cellular components are related to mitochondrial and ribosome pathways (mitochondrial ribosome, mitochondrial matrix, mitochondrial membrane, cytosolic ribosome, and ribonucleoprotein complex). These pathways include the 40S and 60S ribosomal genes, ubiquinone genes such as NADH dehydrogenase, and several mitochondrial enzymatic genes, including superoxide dismutase, pyruvate dehydrogenase, propionyl CoA carboxylase, and isobutyryl CoA dehydrogenase. Finally, GO terms in molecular function are related to signalling receptor and signalling transduction pathways (signalling receptor activity, G protein-coupled receptor activity, and transmembrane signalling receptor activity). Among the signalling receptor pathways, we can find many genes coding for neurotransmitter receptors, like the N-methyl-D-aspartate (NMDA) receptor, dopamine receptor, serotonin receptor (5-HT), gamma-aminobutyric acid (GABA) B receptor, acetylcholine receptor (AChR), FMRFamide receptor, octopamine receptor and orexin receptor. The common enriched metabolic, mitochondrial, and ribosomal pathways suggest that transcripts engaged in cellular metabolic, energy production, and protein synthesis activity are actively regulated in the CNS of snails.

When we look more in depth in the DE genes whose expression is significantly changed at all pairwise comparison, we discovered that the enriched GO terms include biological processes that are related to metabolism of nitrogen compounds, organic substances, macromolecules, and cellular macromolecules. The cellular component GO enrichment highlights the importance of organelle, intracellular organelle, and intracellular anatomical structure. Finally, molecular function GO enrichment includes extracellular matrix structural constituents and protein binding pathway. More specifically, among the most significant DE genes (FRD-corrected *p*-value) we can find several collagens sequences, most of which are down-regulated in old CNS snails compared to young and adult. Two cytochrome P450 genes (CYP2C3 and CYP2U1) have opposite pattern of gene expression, with the CYP2C3 being highly expressed in young snails while the CYP2U1 has its higher expression in old animals. Other highly significant genes include the neuronal growth factor neurotrophin-3-like whose expression is increasing from young to adult to old snail and the signaling molecule rac GTPase-activating protein 1-like that has a pattern of decreasing expression from young to adult to old animals. A table with the DE genes in all pairwise comparison that have a Blast2GO match is provided in Additional Table 4.

To gain further insights into what specific sets of DE genes are changed from young to adult to old snail CNS, we analysed the top100 DE genes used in Fig. 1D and examined their associated GO terms. A complete list of genes, their Log2 fold change value, FDR-corrected *p*-values, descriptions, and GO terms are provided in Additional Table 5. We found that genes significantly increased from young to adult and remain elevated in old animals, including those involved in receptor signalling activity (e.g. NMDA receptor NR1 and NR2, Mollusc insulin-related peptide MIP, and Notch 3 receptors), signalling transduction mechanism (serine/threonine protein kinase TAO1-like TAOK1, protein kinase A PKA, and serine/threonine-protein phosphatase 2A PIPA), synaptic vesicle proteins (e.g. synaptotagmin1 and 4), ion channels (e.g. voltage-gated K^+^ channels Kv2.1a KCNB1), metabolism (pyruvate kinase PKM-like isoform X3 PKM and phosphopractokinase-like PFKM), membrane/membrane bound organelles (e.g. reticulon-3-A like and fat cadherin), transcription and translation (poly [ADP-ribose] polymerase 14-like gene PARP and translation initiation factor eIF-2 EIF2B4), and peptides and peptide enzymes (Titin-like X2 TTN and Peptidase C1-like). Interestingly, there are only a few DE genes that are significantly increased from adult to old animals. These include monooxygenase/oxidoreductase active (CYP2U1), cysteine dioxygenase type (CD01), and endoglucanase E-4 like and A-like. Genes that are highly expressed in young and then significantly decrease in adult and old animals include ECM structure constituents (e.g. collagen 2A1, collagen 1A1, and fibril-forming collagen 2-chatin like). When comparing expression of genes in adult and old, we found that most genes exhibit significantly lower expression in old animals. These include stress and immune factors (dual oxidase 2-like DUOX2, oxidase activity cytochrome P450 CYP10, suppressor of cytokine signalling 2 SOCS2, and heat shock protein 60 HSP 60), Ca^2+^ binding (Ca^2+^/CaM-serine kinase CASK), protein ubiquitin (Myc binding protein MYCBP2 and ubiquitin-protein ligase E3A), and membrane and cellular entity (cadherin, adhesion G-protein coupled receptor L2 like GPCRL2, and disintegrin). Several above mentioned, stress-related genes and their expressions (TPM) in young, adult, and old *L. stagnalis* CNS are shown in Additional Fig. 2.

### KEGG analysis

**Fig. 3:**
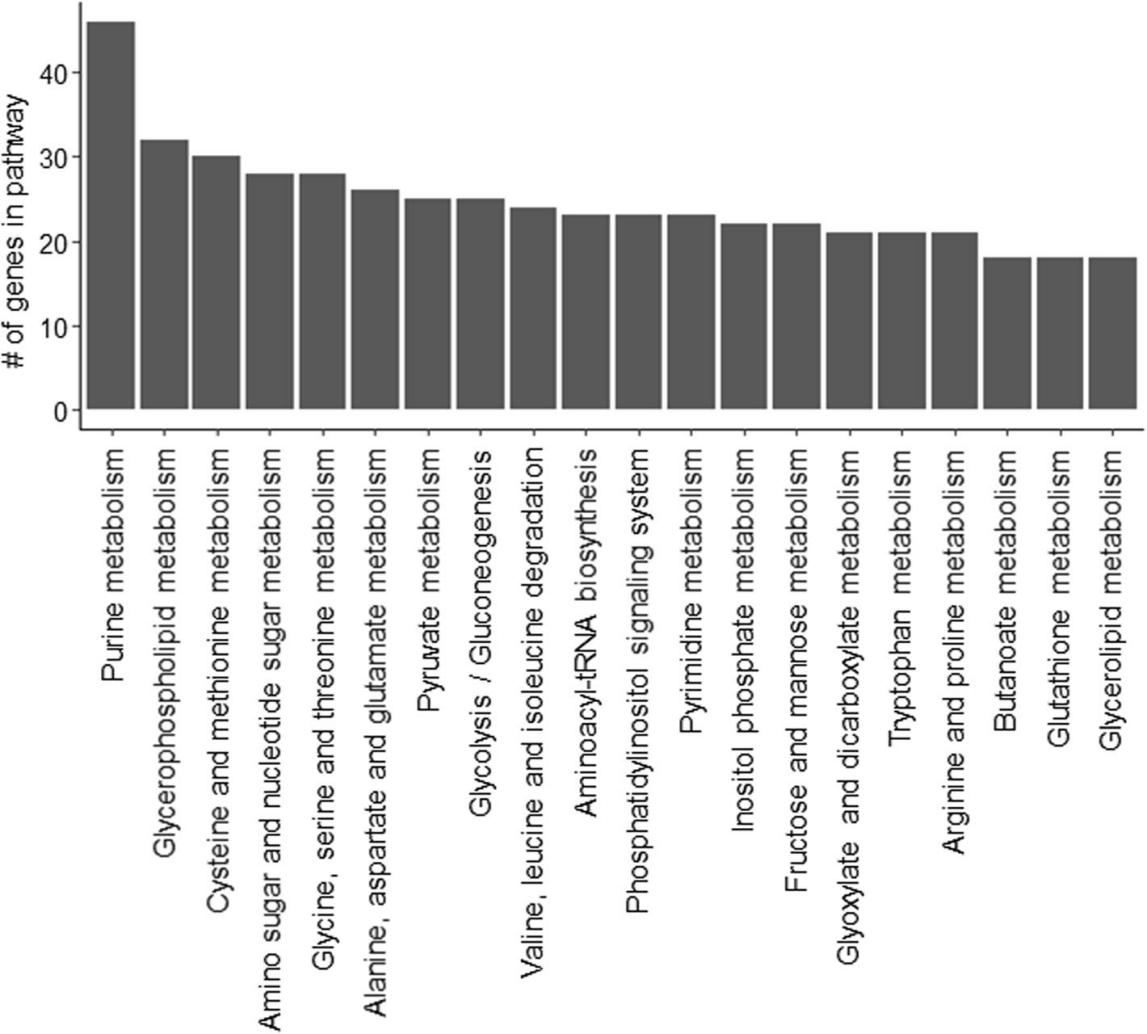
Most representative KEGG pathways in the whole transcriptome. The KEGG pathways associated with our sequences were loaded using Blast2GO. The y-axis shows the number of sequences (or genes) that were annotated in the KEGG pathway. Only the top 20 pathways by number of sequences are shown in the figure. KEGG is developed by Kanehisa Laboratories

Using Blast2GO [51] we also loaded KEGG [52–54] pathways for 37,661 transcripts that encode proteins with at least 100 amino acids (see Methods). A total of 1159 transcripts (or genes) were successfully associated with KEGG pathways. The most representative KEGG pathways in the overall transcriptome include purine metabolism, glycerophospholipid metabolism, cysteine and methionine metabolism, and amino sugar and nucleotide sugar metabolism (Fig. 3**)**. Furthermore, KEGG enrichment analysis was performed for DE genes in: all pairwise comparisons (455 genes), young compared to adult CNS transcriptome (20,141, young compared to old CNS transcriptome (18,394), and adult compared to old CNS transcriptome (3108) (FDR adjusted *p*-value *p* < 0.05; log2 fold change > |1|). The all pairwise comparison and adult compared to old comparison produced significant enrichment for the pathways of thiamine metabolism (all *p* = 2.38 × 10^− 5^, adult vs old *p* = 0.00) and purine metabolism (all *p* = 0.01, adult vs old *p* = 0.06).

### qPCR studies confirm differential gene expression identified by RNA-Seq data

**Fig. 4:**
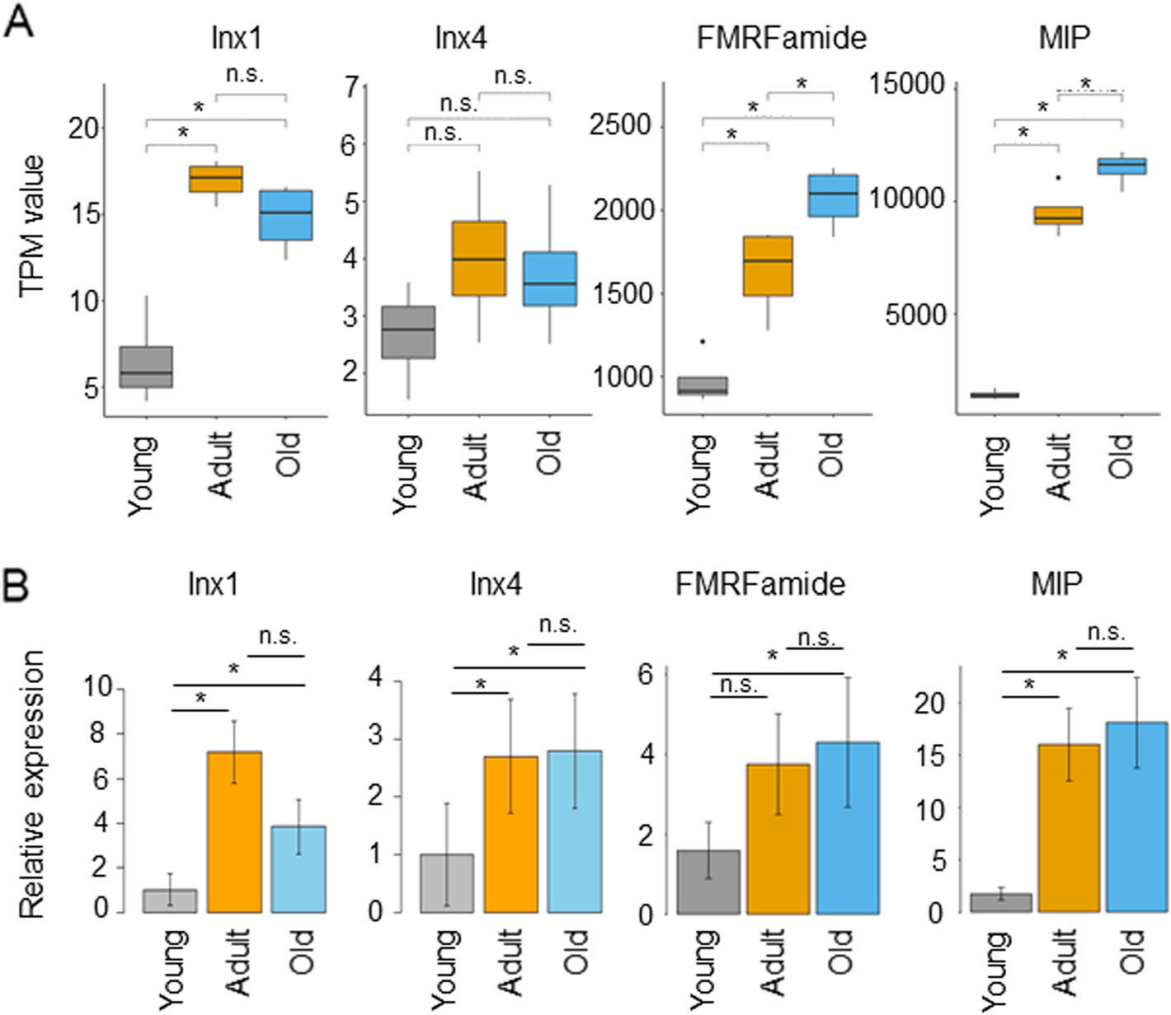
Confirmation of differential gene expression by real-time qPCR of *L. stagnalis* genes. **A** RNA-Seq data reveals gene expression for innexins1/4, FMRFamide, and molluscan insulin peptide (MIP) in the CNS of young, adult, and old snails. **B** real-time qPCR data show patterns of expression comparable to RNA-Seq data. These data show a general concordance of gene expression measured in our transcriptome data compared to qPCR data. * *p* < 0.05

We next performed qPCR to validate gene expression revealed by RNA-Seq for a few genes that are previously cloned by us (i.e. gap junction-forming innexin genes) [17] or others (i.e. FMRFamide and *MIP* genes) [55–57]. As shown in Fig. 4A,B, both RNA-seq data and qPCR data show a similar pattern of expression over ages for each gene. More specifically, *Inx1* has a significantly lower expression level in young animals compared to adult and old (RNA-Seq FDR adjusted *p*-value young vs adult *p* = 5.95 × 10^− 7^, young vs old *p* = 3.73 × 10^− 6^; qPCR Turkey’s post-hoc test young vs adult *p* = 0.00 young vs old *p* = 0.00). *Inx4* also has similar trends in gene expression in both RNA-Seq and qPCR, but it is expressed significantly lower in the young animals only in the qPCR data (RNA-Seq FDR adjusted *p*-value young vs adult *p* = 0.39, young vs old *p* = 0.39; qPCR Turkey’s post-hoc test young vs adult *p* = 0.00, young vs old *p* = 0.00). Similar to *innexin* genes, both RNA-Seq and qPCR data for FMRFamide and *MIP* genes show comparable pattern of expression over ages. More specifically, both *MIP* and *FMRFamide* show significant increase in expression from young to adult or old snails (*MIP* RNA-Seq FDR adjusted *p*-value young vs adult *p* = 5.88 × 10^− 86^, young vs old *p* = 5.79 × 10^− 119^, qPCR Turkey’s post-hoc test young vs adult *p* = 0.00, young vs old *p* = 0.00; *FMRFamide* RNA-Seq FDR adjusted *p*-value young vs adult *p* = 0.03, young vs old *p* = 0.00; qPCR Turkey’s post-hoc test young vs adult *p* = 0.10, young vs old *p* = 0.04). Overall, the concordance between RNA-Seq transcriptome expression and qPCR confirms the reliability of our RNA-Seq measurements.

### Quality estimate of transcription assembly: coverage of previously cloned genes

To further evaluate the quality and completeness of the transcriptome assembly, we tested the coverage of previously cloned genes from *L. stagnalis* in our transcriptome. According to the most recent NCBI nucleotide database, a total of 242 *L. stagnalis* genes have been previously cloned (Additional Table 1). There are 210 of these genes (87%) present in our transcriptome assembly, supporting that our transcriptome assembly from CNS samples cover most of the previously known protein-coding genes in *L. stagnalis*. Considering that only a portion of genes are expressed in brain tissues, it is expected that some previously cloned genes would not be detected by this RNA-Seq study.

**Fig. 5:**
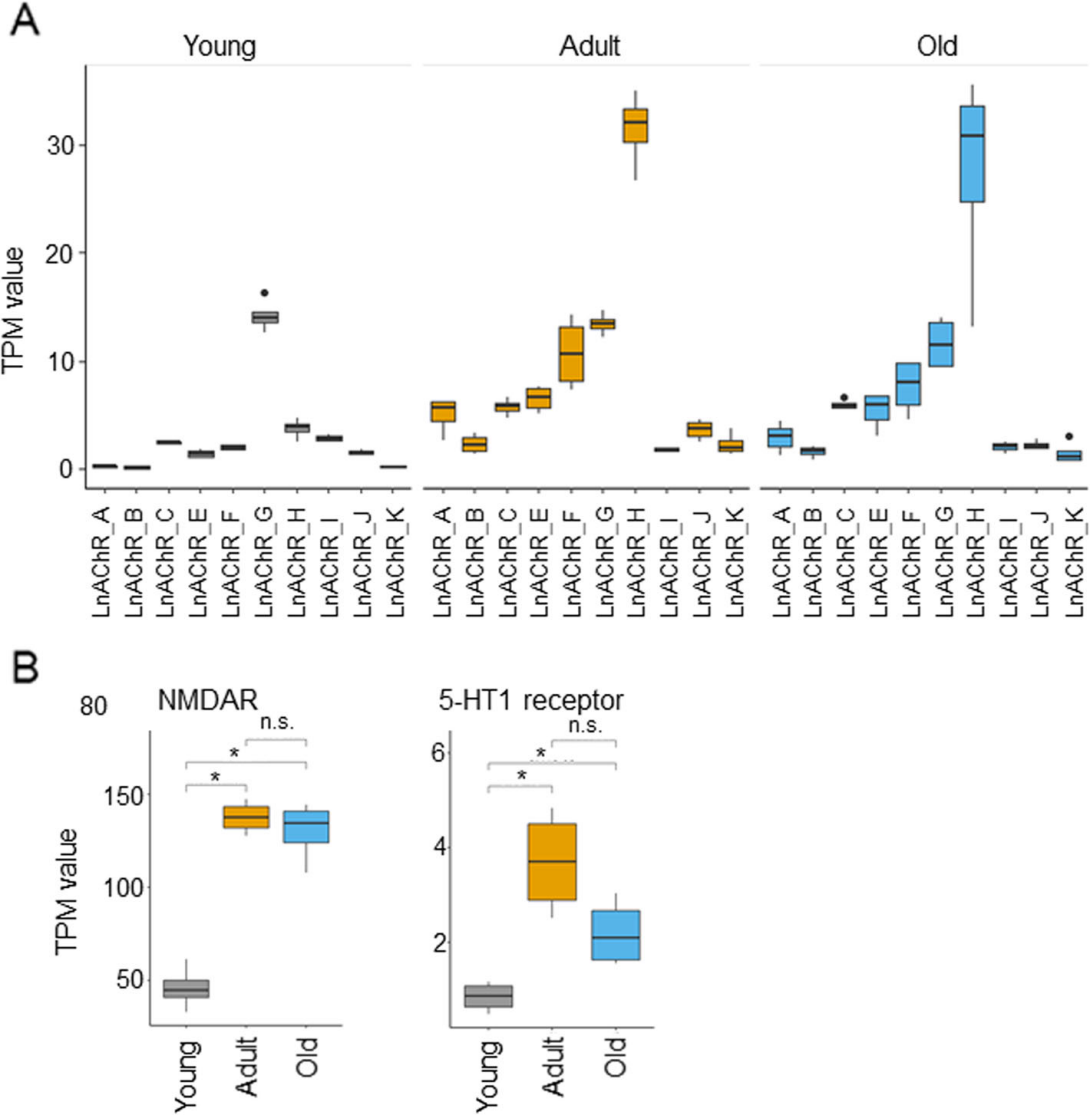
Differential expression of previously cloned synaptic receptor genes in *L. stagnalis*. **A** TPM expression of different *L. stagnalis* acetylcholine receptor subunits (LnAChR). Consistent with previous literature, the most highly expressed subunit in adult CNS transcriptome is the subunit H. Old snail CNS transcriptome has a similar pattern of LnAChR expression as adult snails. In young snails, though, the most highly expressed subunit is G. These data suggest that acetylcholine receptor subunits are specifically expressed at different life stages. **B** Genes involved in synaptic transmission (NMDAR, N-Methyl-D-aspartic acid receptor; 5-HT receptor, and serotonin receptor) are significantly downregulated in young snails CNS compared to adult and old. The different patterns of expression for neurotransmitter receptors in young versus adult and old snails suggest that CNS synaptic development requires specific patterning to establish functional synapses throughout the span of *L. stagnalis* life. * *p* < 0.05

Among the previously cloned genes mapped in our transcriptome, we can find almost all of the *L. stagnalis* acetylcholine receptor (LnAChR) subunits. Of the twelve subunits, we found ten in our transcriptome. We further show that in the CNS transcriptome of adult and old snails, the LnAChR subunits H and F have the highest expression [58]. Interestingly, in the CNS transcriptome of young snails, subunit G has the highest expression (Fig. 5A). These data seem to suggest changes in LnAChR composition during CNS development. Moreover, two other synaptic receptors, the NMDAR and the serotonin receptor (5-HT receptor) have a significantly lower expression in the young compared to adult and old CNS transcriptomes (NMDAR: RNA-Seq FDR adjusted *p*-value young vs adult *p* = 6.35 × 10^− 16^, young vs old *p* = 3.08E-17; 5-HT receptor: RNA-Seq FDR adjusted *p*-value young vs adult *p* = 9.90 × 10^− 10^, young vs old *p* = 9.41 × 10^− 5^) (Fig. 5B). Together, these data suggest that our transcriptomes indeed reveal the expression of many previously cloned genes and their subunits.

### Changes in expression of disease-related genes in young, adult and old animals

Lastly, a recent paper using both *A. californica* and *L. stagnalis* has provided the cloned sequences for several genes involved in neurodegenerative disorders like Huntington disease, Parkinson’s disease (PD), and Alzheimer’s disease (AD) [24]. Identification and expression analysis of these genes in the *L. stagnalis* transcriptome is promising for the use of *L. stagnalis* as a model for studying neurodegenerative diseases. In our transcriptome, we discovered that the expression of these genes changes with age. More specifically, Parkinson’s disease protein 7 (*PARK7*), huntingtin, choline acetyltransferase, and presenilin genes are all upregulated in the CNS of adult and old compared to young animals (Additional Fig. 3).

In addition to these well-recognized genes, there are several other genes that are changed in adult and old animals compared to young animals. These include Arginase 1 (ARG1, linked to many human diseases), reticulons (linked to AD and amyotrophic lateral sclerosis) [59], proton myo-inositol cotransporter (SCL2A13, linked to AD) [60], Rab GDP dissociation inhibitor (linked to mental retardation) [61], and several tumor genes such as tumor protein D52 (TPD 52), tumor suppressor gene e-cadherin like, and protein phosphatase2A (PP2A); a few of these genes and their expressions in young, adult, and old *L. stagnalis* are shown in Additional Fig. 3. Expression of these disease-related genes in *L. stagnalis* provides a unique opportunity for using *L. stagnalis* as a model system to study these genes.

## Discussion

*L. stagnalis* has served as a unique model organism for the study of neural networks, neuronal development, and synapse formation [6, 14, 16] due to its simple, well-characterized, and easily accessible CNS. In addition, it has recently emerged as a useful model for studying brain aging and neurodegenerative diseases [22–24]. Here, we generated datasets that allow for the first in-depth look at transcriptome changes in gene expression of *L. stagnalis* CNS from young (3 month), adult (6 month), and old (18 month) animals. Our study identifies new *L. stagnalis* sequences, with a good read depth of up to 69 million total fragments (150 bp paired-end reads); moreover, we took advantage of the Blast2GO bioinformatics platform to provide gene annotation and gene ontology (GO) annotation for over 30,000 sequences. This study also reveals temporal dynamics of transcriptional profiling and key DE genes/pathways in *L. stagnalis* CNS at multiple time points of the animal’s life span. Such information will be instrumental for future age-related phenotypical analyses in single cells, neuronal networks, and whole animals.

Knowledge about age-associated transcript changes can improve our understanding of how intrinsic profiling plays a role in influencing anatomical, physiological, and pathophysiological properties in animals and human at different life stages. Transcriptomic analyses of human brains at different ages have shown that the majority of protein-coding genes are spatiotemporally regulated, and the transcriptional differences are most pronounced during early development [33]. Similarly, our data reveal that constitutive differences in transcriptomes exist between young and adult CNS, while adult and old CNS exhibit fewer differences in transcripts. In the rat hippocampus, 229 genes were reported to be linearly up- or down-regulated across the lifespan of a healthy animal [35]. Previous studies of transcriptomic profiling of sensory neurons from *A. californica* at 8 (matured), and 12 (aged) months reported that half of the genes were up- and half of the genes were down-regulated between the two cohorts of neurons [48]. Our data in this study demonstrates that in *L. stagnalis* CNS, there are genes exhibiting linear up- or down-regulation from young to adult to old (Fig. 1D), but there are also many genes that are regulated in a non-linear manner. For example, some genes are upregulated from young to adult and appear to either maintain a comparable level of expression or significantly decreased expression in old animals as shown in Figs. 4-5 and Additional Fig. 2-3**.** The linear or non-linear expression of genes across animals at different ages and in different species may highly correlate with age- and species-specific functions of these genes. Interestingly, the principal component analysis revealed that the majority of *L. stagnalis* (Additional Fig. 1) biological replicates clustered together. The same clustering pattern was found in *A. californica* [48] suggesting that individuals of these two species in the same age group share similar transcript profiles. These results suggest that age is an important, determinizing factor for transcriptional profiling between individuals. Together, these data support that whole transcriptome comparison can serve as a valuable tool for discovering age-specific genes, and these mollusc organisms could serve as useful models for studying age-related molecular basics of brain development and aging.

Our analyses indicate that the majority of DE genes occur between young and adult CNS, and the DE genes are enriched in the GO pathways related to metabolic processes, gene expression, mitochondrial, and ribosome, as well as signalling receptor pathways. Further KEGG analysis revealed significant regulation of pathways for thiamine metabolism and purine metabolism, which regulate energy and nucleotide availability, respectively. These findings are consistent with a recent study of adult *L. stagnalis* CNS transcriptome compared to several other adult organisms used in neurobiology including *Mus musculus* (mouse), *D. rerio* (zebrafish), *Xenopus tropicalis*, *C. elegans,* and *D. melanogaster* [31]. Specifically, this study focused on the annotation of the top 20 expressed transcripts in these models. The authors revealed an abundance of transcripts involved in energy production, protein synthesis, and signalling transduction of adult CNS, indicating evolutionally conserved roles of these pathways in mature adult animals of both invertebrates and vertebrates. Because of the importance of these pathways in adult animals, it is not surprising that previous studies have primarily focused on cloning genes involved in these pathways. Indeed, among many cloned genes in our *L. stagnalis* transcriptome, we can detect DE genes encoding proteins involved in these pathways. For example, our RNA-Seq data identified ten of the twelve *L. stagnalis* LnAChR subunits that have been previously cloned and sequenced [58]. Interestingly, we provide evidence that these subunits are differentially expressed in the snail’s CNS at different ages: the subunit H is most highly expressed in adult and old, followed by the F subunit, while the G subunit is most highly expressed in young snails. The expression of LnAChR subunits in adult snails is consistent with literature showing that subunits H and F together account for approximately half of LnAChR expression in the *L. stagnalis* CNS as shown by in situ hybridization (ISH) [58]. Studies in rat brain have also shown that various nAChR subunits are expressed at different ages and in different brain areas [62, 63]. Studies comparing primates and humans to rodent brains have shown that the expression of nAChR subunits is conserved in some brain areas but not others [64]. Furthermore, molluscan and other invertebrate species are known to have not only excitatory, sodium-selective nAChRs, but also inhibitory, chloride-selective nAChRs [65]. Considering the properties of cation or anion conductance of LnAChR subunits, we can appreciate the importance of differential expression of these subunits across the lifespan of *L. stagnalis* for maintaining excitability homeostasis*.* Specific pharmacological properties have been demonstrated for nAChRs composed of different subunits in both mammalian [64, 66], and invertebrate models [67]. Our transcriptome data suggest that the expression pattern or properties of LnAChRs might be important in CNS development and function. Therefore, it would be interesting to investigate the pharmacological properties of the different LnAChR subunits and their spatiotemporal expression and function in the CNS of *L. stagnalis* in the future.

Among other previously cloned neurotransmitter receptors, we found that the N-Methyl-D-aspartic acid (NMDA) and the serotonin (5-HT) receptors are differentially expressed when comparing the CNS of young and adult/old snails (Fig. 5B). Studies in both human [68, 69] and rat [70] have shown differential expression of the NMDAR subunits NR1 and NR2 at different ages. Similarly, 5-HT receptors have been shown to be differentially expressed during human early postnatal development and into adulthood [68, 71]. Importantly, aberrant expression and/or function of NMDA and 5-HT receptors have been associated with neurodevelopmental disorders such as schizophrenia and autism [72–77]. These changes in neurotransmitter receptor expression at different ages suggest ongoing synaptic development or synaptic diversification when *L. stagnalis* CNS progresses from young to a fully matured stage. The significant up-regulation of genes encoding these transmitter receptors as well as synaptotagmin, gap junctions, ion channels, FMRFamide and MIP (Additional Table 4 and results) clearly indicates the active engagement of intercellular communication and synaptic plasticity in these adult animals. Transcript regulation of synaptic genes may reflect animal behavioural changes; compared to young, adult and old animals normally exhibit more vigorous and diverse behaviours including reproduction, feeding, locomotion, respiration, and associative learning, for which the above-mentioned synaptic machinery components play major roles [5, 17, 31, 78–82].

In addition to previously known genes, it is interesting to note a few unstudied genes in the *L. stagnalis* transcriptome that exhibit age-specific expression patterns across life stages (as described in results). For example, genes related to oxidative stress and immunity response are either up- or down-regulated in old animals when compared to young and adult animals (Additional Fig. 2). These include cytochrome P450 (CYP2U1 and CYP10), dual oxidase 2 (DUOX2), and suppressor of cytokine signalling 2 (SOCS2). The cytochromes P450 (CYPs) constitute a large superfamily of hemeproteins that are largely involved in the oxidative metabolism of environmental (xenobiotics such as drugs and pesticides) or endogenous (steroid hormones, fatty acid, etc) compounds [83, 84]. Both CYP2U1 transcripts and proteins are widely expressed in various brain regions of human and rats and are involved in the metabolism of fatty acids and xenobiotics in the brain [83]. Interestingly, CYP10 has been cloned in *L. stagnalis* and found to be abundantly expressed in the female gonadotropic hormone producing dorsal bodies [85]. DUOX are oxidoreductase enzymes that catalyse the synthesis of reactive oxygen species (ROS) molecules including the anion superoxide (O_2_^−^) and hydrogen peroxide (H_2_O_2_). DUOX as well as the previously cloned GPX (Supplemental Fig. 2) have recently been demonstrated to be DE in *L. stagnalis* transcriptome in response to ecoimmunological challenges [86]. SOC2 acts as a negative feedback inhibitor for a variety of cytokine signalling in both vertebrates and invertebrates [87, 88]. Because of the significant regulation of these oxidative stress and immune defense genes across life stages, it is critical to study their roles in animal health, aging, and diseases in future studies.

Finally, our transcriptomic data also revealed changes in disease-relevant genes associated with neurodegeneration, aging, and cancer, thus affording a unique opportunity to study cellular and molecular functions of these genes by taking advantage of the simplicity of *L. stagnalis* CNS. In addition, sequenced homologs of several genes known to be involved in aging and neurodegenerative disease (e.g. Parkinson’s disease protein 7 (*PARK7*), huntingtin, presenilin1, and choline acetyltransferase (AChAT) [24], among others) have recently become available. Our data reveals that in *L. stagnalis,* the expression of these genes is upregulated in adult and old CNS compared to young CNS. In addition to these genes, in the present study, we have discovered several other disease-related genes (Additional Table 5 and Additional Fig. 3). Firstly, the membrane proton myo-inositol cotransport (SLC2A13) increases expression in the CNS of adult and old compared to young *L. stagnalis*. Similar to presenilin1, proton myo-inositol cotransport is found to be a novel gamma-secretase associated protein that selectively regulates Aβ production [60]. Secondly, the present study reveals increased expression of reticulons which have been linked to AD and amyotrophic lateral sclerosis (ALS) [59]. Reticulons is a group of evolutionarily conserved proteins residing predominantly in the endoplasmic reticulum that promote membrane curvature, vesicle formation, and nuclear pore complex formation. Since all these proteins are potentially related to AD, it would be interesting to investigate their roles in learning and memory or aging in future studies. While mutation, deletion, or decrease in expression of these disease-related genes are the primary cause of diseases [89–92], the purpose for maintaining a high expression of these gene transcripts in the CNS of adult and old animals is not known. However, our results may partially indicate that the abundant expression of these disease-related genes could be a result of normal physiological requirements or the natural aging process of mollusc CNS.

## Conclusions

Overall, our RNA-seq study provided a much-needed *L. stagnalis* transcriptome assembly, with gene and GO annotation for more than 30,000 predicted genes. Furthermore, the analysis of CNS from different ages demonstrates the importance of this model for uncovering molecular insights in young, adult, and old life stages. This dataset will be useful for future discoveries of genes, expression profiling, and signalling pathways in different ages of animals. It also serves as a helpful resource for future annotation of genes and the genome of *L. stagnalis*.

## Methods

### Animals and brain dissection

*L. stagnalis* were maintained in artificial pond water at 20 °C in a 12-h light/dark cycle and fed with romaine lettuce twice a week. *L. stagnalis* were obtained from the University of Calgary, Canada (original stock was from the Vrije University in Amsterdam) and raised and maintained in aquaria at Saint Louis University since 2015 according to protocols developed and optimized as described previously [17, 91]. All procedures are in accordance with the standard operating protocol guidelines established by the U.S. Department of Agriculture Animal and Plant Health Inspection Service. Animals at 3 months old (young), 6 months old (adult) or 18 months old (old) were used for RNA-Seq and qPCR. We used four replicate samples for each developmental age, and for each sample, the CNS of ten animals were pooled. The snails were de-shelled and anesthetized in 10% (v/v) Listerine in *L. stagnalis* saline (51.3 mM NaCl; 1.7 mM KCl; 4.0 mM CaCl_2_; 1.5 mM MgCl_2_, 10 mM HEPES, pH 7.9), and the dissected central ring ganglia were used for both RNA-Seq and qPCR.

### RNA extraction

RNA was extracted from dissected *L. stagnalis* central ring ganglia using the RNeasy Mini Kit (Qiagen, 74,104) following the manufacturer’s instructions. RNA concentration was assessed using a Nanodrop 2000 Spectrophotometer (ThermoFisher, ND-2000). After RNA extraction, genomic DNA (gDNA) was removed via the TURBO DNA-free kit (Invitrogen, AM1907).

### RNA sequencing library construction, sequencing, alignment, and transcriptome assembly

The construction of RNA-Seq libraries using polyA enrichment method was performed by Novogene Corporation Inc. (Sacramento, CA, USA). These libraries were sequenced using the Illumina NovaSeq 6000 platform (Paired-end, 150 bp, insert size 250–300 bp). The sequencing reads of each RNA-Seq library were aligned to the reference genome of *L. stagnalis* (assembly v1.0 GCA_900036025.1) using HISAT2 [92]. The soft clipping option in HISAT2 was enabled to exclude low-quality bases at both ends of reads. The number of reads in each sample successfully mapped to the *L. stagnalis* reference genome are provided in Table 1. We used Stringtie (93) to assemble transcripts based on aligned reads. The expression abundance of each transcript was quantified as fragment per kilobase million reads (FPKM). To provide between-samples comparability, the data is presented as transcripts per million (TPM) in the figures. The results of principal component analyses (PCA) of the abundance of transcripts (FPKM) for all genes from all samples are provided in Additional Fig. 1A**.** The correlations of gene expression profile between each pair of samples are provided in Additional Fig. 1B. The raw sequencing data generated in this study have been submitted to the NCBI BioProject database under accession number PRJNA698985.

### Functional annotation of inferred *L. stagnalis* genes

We first used TransDecoder v5.5.0 (94) to retrieve CDS and amino acid sequences for each assembled transcript from the *L. stagnalis* reference genome based on the merged annotation file generated by Stringtie. We applied two different methods to annotate inferred *L. stagnalis* genes and combined the annotated information. The first method was based on BLASTP searches against NCBI RefSeq amino acid sequences of nine species closely related to *L. stagnalis*. These species included: *Biomphalaria glabrata*, *Aplysia californica,*, *Lottia gigantea*, *Pomacea canaliculata*, *Octopus bimaculoides*, *Octopus vulgaris*, *Crassostrea virginica*, *Crassostrea gigas*, and *Mizuhopecten yessoensis*. The second method was to search for the presence of Pfam domains in the inferred *L. stagnalis* amino acid sequences using the “hmmscan” tool in HMMER3 (95). The BLASTP and Pfam search results were integrated into the annotation of predicted *L. stagnalis* open reading frames (ORFs) using TransDecoder-v5.5.0 (94).

### Gene ontology and KEGG annotation of inferred genes in *L. stagnalis*

We used predicted protein sequences of *L. stagnalis* with at least 100 amino acids for both Gene Ontology (GO) and KEGG [52–54] annotation using Blast2GO [50]. This annotation analysis was based on homology searches against the Mollusca phylum, *C. elegans*, *D. melanogaster*, and *H. sapiens* using the latest reference protein database (refseq_protein v5). We used an e-value threshold of 1.0E-3, top 20 blast hits, word size 6, and HSP length cut-off of 33. GO annotation was based on the latest GO version (2020.06). For both GO and KEGG enrichment analysis, Fisher’s exact test was used in combination with a False Discovery Rate (FDR) correction for multiple testing (FDR < 0.05). R (96) package ggplot2 was used to plot results of both enrichment analyses.

### Identification of previously cloned genes in *L. stagnalis*

We searched for previously cloned genes in *L. stagnalis* from the NCBI nucleotide database (as of January 2021) to evaluate the completeness of our transcriptome assembly. Only cloned genes that were supported by published studies were selected. The list of previously cloned genes in *L. stagnalis* (NCBI ID, gene names and references) is provided in Additional Table 1.

### Differential gene expression analysis and validation of gene expression by real-time qPCR

Differential expression (DE) analysis was carried out using DESeq2 (97) based on raw read counts retrieved by the featureCounts package of Subread v1.5.0 (98). The results of DE analysis by DESeq2 are shown in Additional file 1.

We validated the differential gene expression through real-time quantitative polymerase chain reactions (RT-qPCR). cDNA synthesis was performed from gDNA-removed RNA samples using SuperScript IV VILO Master Mix (Invitrogen, 11,766,050) following the manufacturer’s instructions. SYBR Green PCR Master Mix (Applied Biosystems, 4,309,155) was used for RT-qPCR in a QuantStudio 5 Real-Time PCR System (ThermoFisher). Primers are listed in Additional Table 2. Primer set efficiency values ranged from 95.85–104.26%, and R^2^ values were 0.98-0.99. Two negative controls were used: qPCR without reverse transcription and no template controls. Relative gene expression was normalized to reference gene *β-tubulin*. The final qPCR product was also sequenced to ensure the correct *innexin* paralog was amplified.

Because of the wide range of primer efficiencies, relative gene expression was calculated via the Common Base Method (99) and normalized to reference gene *β-tubulin*. Analysis of variance (ANOVA) was used to determine statistically significant differences in gene expression at *p* < 0.05, and Tukey’s HSD *Post-hoc* test was used when appropriate.

## Supplementary Information

**Additional file 1.**

**Additional file 2.**

**Additional file 3.**

**Additional file 4.**

**Additional file 5.**

**Additional file 6.**

**Additional file 7.**

**Additional file 8.**

**Additional file 9.**

**Additional file 10.**

## Data Availability

All raw RNA-seq reads are archived under NCBI BioProject # PRJNA698985 (https://www.ncbi.nlm.nih.gov/bioproject/?term=PRJNA698985). Previously cloned gene list and NCBI accession numbers are provided in Additional Table 2. Annotation and transcript sequences information are provided in Additional files 1 and 2.

## Abbreviations

CDS: Coding sequences
CNS: Central nervous system
EST: Expressed sequence tags
DE: Differentially expressed
GO: Gene ontology
FPKM: Fragment per kilobase million
TPM: Transcripts per milion
NMDA: N-methyl-D-aspartate receptor
CYP: Cytochrome P450
CDO1: Cysteine dioxygenase 1
ECM: Extracellular matrix
DUOX2: Dual oxydase 2
SOCS2: Suppressor of cytokine signaling 2
HSP60: Heat-shock protein 60
LnAChR: *L. stagnalis* acetylcholine receptor
5-HT: Serotonin
MIP: Molluscan insulin peptide
AQP1: Aquaporin1
TPH: Tryptophan hydroxylase
GPX: Glutathione peroxidase
PD: Parkinson’s disease
AD: Alzheimer’s disease
PARK7: Parkinson’s disease protein 7
ChAT: Choline acetyltransferase
ARG-1: Arginase 1
SCL2A13: Membrane proton myo-inositol cotransporter
PP2A: Protein phosphatase 2A
