# Supplementary figures and images for: Transcriptome analysis provides genome annotation and expression profiles in the central nervous system of Lymnaea stagnalis at different ages
Source: BMC Genomics. 2021 Sep 3;22:637. doi: 10.1186/s12864-021-07946-y (PMC8414863; doi:10.1186/s12864-021-07946-y)

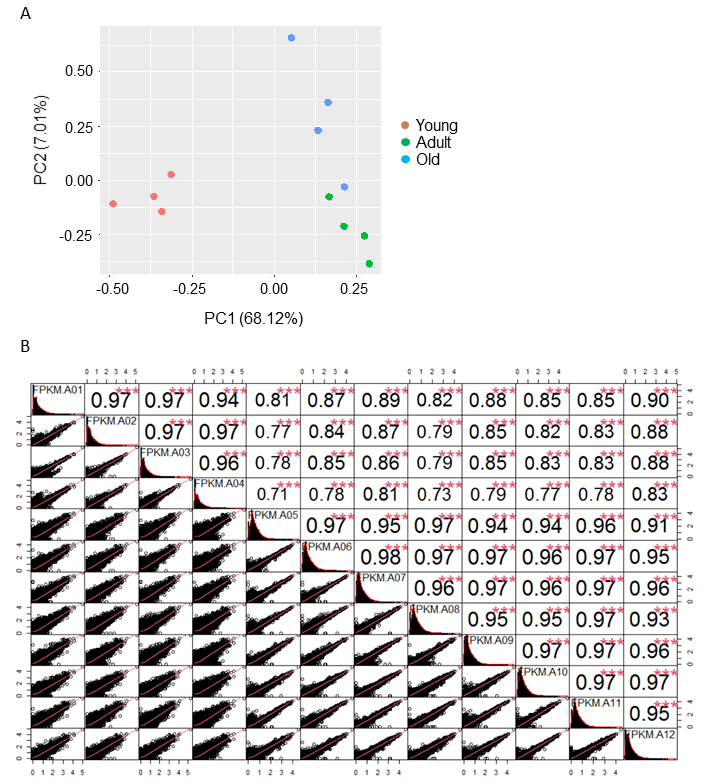

Supplement: Supplementary file 8 — Additional file 8. [file 12864_2021_7946_MOESM8_ESM.tif]

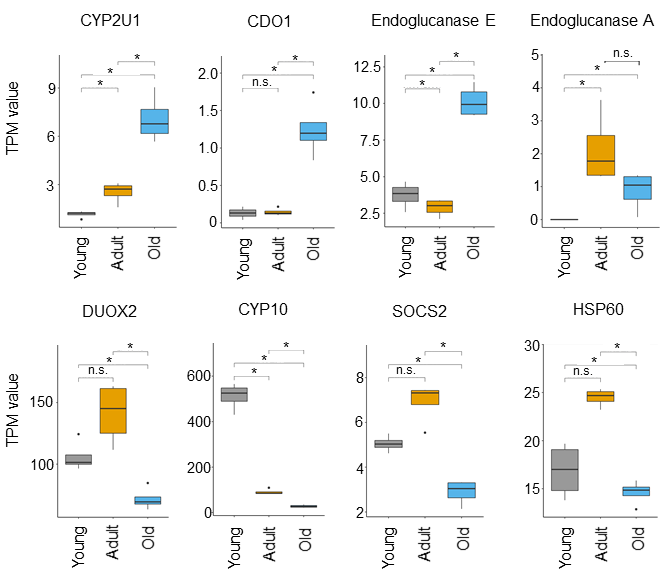

Supplement: Supplementary file 9 — Additional file 9. [file 12864_2021_7946_MOESM9_ESM.tif]

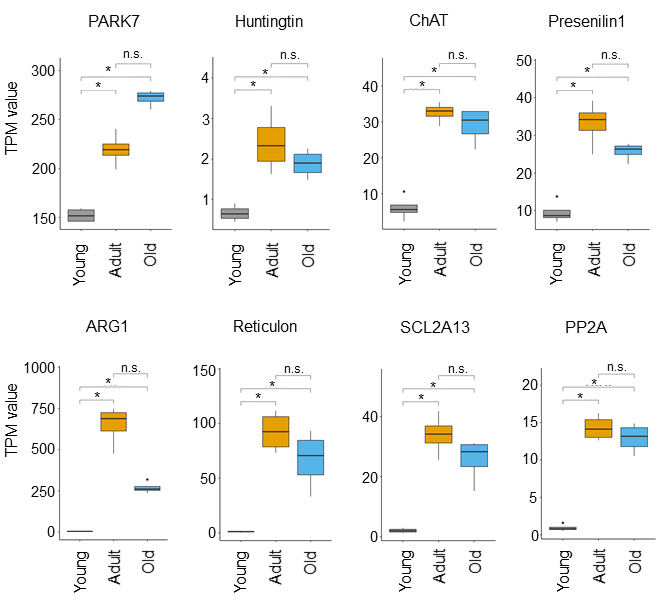

Supplement: Supplementary file 10 — Additional file 10. [file 12864_2021_7946_MOESM10_ESM.tif]
